# Radio-frequency exposure of the yellow fever mosquito (*A*. *aegypti*) from 2 to 240 GHz

**DOI:** 10.1371/journal.pcbi.1009460

**Published:** 2021-10-28

**Authors:** Eline De Borre, Wout Joseph, Reza Aminzadeh, Pie Müller, Matthieu N. Boone, Iván Josipovic, Sina Hashemizadeh, Niels Kuster, Sven Kühn, Arno Thielens

**Affiliations:** 1 Ghent University - Imec, Department of Information Technology, Ghent, Belgium; 2 Swiss Tropical and Public Health Institute, CH-4002 Basel, Switzerland; 3 University of Basel, Basel, Switzerland; 4 Centre for X-ray Tomography (UGCT), Department of Physics and Astronomy, Ghent University, Ghent, Belgium; 5 Foundation for Research on Information Technologies in Society (IT’IS), Zurich, Switzerland; University of Tokyo: Tokyo Daigaku, JAPAN

## Abstract

Fifth generation networks (5G) will be associated with a partial shift to higher carrier frequencies, including wavelengths comparable in size to insects. This may lead to higher absorption of radio frequency (RF) electromagnetic fields (EMF) by insects and could cause dielectric heating. The yellow fever mosquito (*Aedes aegypti*), a vector for diseases such as yellow and dengue fever, favors warm climates. Being exposed to higher frequency RF EMFs causing possible dielectric heating, could have an influence on behavior, physiology and morphology, and could be a possible factor for introduction of the species in regions where the yellow fever mosquito normally does not appear. In this study, the influence of far field RF exposure on *A*. *aegypti* was examined between 2 and 240 GHz. Using Finite Difference Time Domain (FDTD) simulations, the distribution of the electric field in and around the insect and the absorbed RF power were found for six different mosquito models (three male, three female). The 3D models were created from micro-CT scans of real mosquitoes. The dielectric properties used in the simulation were measured from a mixture of homogenized *A*. *aegypti*. For a given incident RF power, the absorption increases with increasing frequency between 2 and 90 GHz with a maximum between 90 and 240 GHz. The absorption was maximal in the region where the wavelength matches the size of the mosquito. For a same incident field strength, the power absorption by the mosquito is 16 times higher at 60 GHz than at 6 GHz. The higher absorption of RF power by future technologies can result in dielectric heating and potentially influence the biology of this mosquito.

## Author summary

Radio Frequency (RF) exposure of the *A*. *aegypti* mosquito can lead to absorption and dielectric heating. We used Finite Difference Time Domain (FDTD) simulations between 2 and 240 GHz to study the RF power absorbed by the insect and the distribution of the electric field (EF) in and around it. For this, three male and three female mosquito 3D models were constructed from micro-CT scans. We used high resolution models and dielectric properties, both retrieved from real insects, to gain realistic outputs. For increasing frequency up to 90 GHz, the absorbed power increases for all models. At 90–120 GHz, the wavelength is comparable to the body size, and the increase in absorbed powers reaches a maximum. Therefore, moving to higher frequencies in 5G, implies higher absorbed power and possibly higher dielectric heating of the insect.

## Introduction

With the upcoming fifth generation networks (5G) in wireless telecommunications, the Radio Frequency (RF) electromagnetic fields (EMF) used as carriers will partly shift to higher frequencies. Current telecommunication networks make use of frequencies of 0.1–6 GHz [1], while the carrier frequencies for 5G networks can go up to 300 GHz, entering the millimeter-wave frequency range [2]. For these higher frequencies, the wavelength becomes comparable to the body size of insects. When wavelength and body size become of the same order of magnitude, an increase in efficiency of absorption of RF-EMFs in the body is expected [3]. The absorption of RF-EMF in biological tissues can lead to the dielectric heating of an organism [4]. With the alternating electric field and polarity, the movement of free ions and dipoles causes the heating in dielectric material [5]. This RF heating of insects has been repeatedly investigated as a method to kill insects in low-moisture foods, grain, or wheat storage [6–13]. The difference in dielectric properties, RF absorption and consequential dielectric heating between insect and food, is used to heat the insect up to a lethal temperature for the insect, while the food is not damaged. The frequency and field strength are chosen depending on the type of stored food and the infesting insect.

The insect of interest in this paper is the yellow fever mosquito, *Aedes aegypti*, it is known as a vector for diseases such as yellow fever, dengue fever and zika virus infections [14, 15]. According to the Centers of Disease Control and Prevention in the U.S., yellow fever cases and deaths worldwide are estimated at 200,000 and 30,000 each year [16], respectively. The yellow fever mosquito is a tropical species favouring a hot and humid environment. Temperature affects the life cycle and feeding behaviour of the mosquito and the reproduction of the viruses [14, 15]. RF power absorption and dielectric heating can cause disturbance in for example the behaviour or development of the mosquito. Another interesting consequence of dielectric heating and higher body temperature, may be the spread of the mosquito to areas that are normally unfavorable for them. Be that as it may, the focus in this paper is on the RF power absorption, the dielectric heating or other consequences are not considered.

The absorbed RF power in four different insect species has been examined through numerical simulations by Thielens *et al*. using models created from real insects [17, 18] and it was observed that absorbed power was maximal for wavelengths comparable to the insects’ body size. However, the simulations in [17, 18] relied on unverified assumptions regarding the dielectric properties of the studied insects and these arthropods were at least a factor 1.5 larger than the yellow fever mosquito in terms of volume.

The effects of RF-EMF exposure can have an important impact on insects, they have been investigated experimentally on several insects. Influences were reported on e.g. the development and mating of honeybees (reduced hatching of honey bee queens) [19], behaviour of ants (the locomotion) [20], and the morphology of mealworm beetles during development (abnormalities of appendages) [21]. The collective position behaviour of mosquitoes *A*. *aegypti* was examined under relatively low-power RF-EMFs in the frequency range of 0.01–20 GHz, without any conclusive results about the position of the mosquitoes as a reaction to the RF-EMF exposure [22]. The absorption of RF-EMFs of mosquitoes has however never been investigated, nor their dielectric properties.

The dielectric parameters, relative permittivity (*ϵ*_*r*_) and conductivity (*σ*), have been characterized for other insects before. Coaxial-probe measurements were done on stored-grain insects [23], the Colorado Potato Beetle [24], and insects in dried nuts and fruits [6]. An alternative resonance method has been used as well to investigate dielectric properties of different insects [25].

In this paper, the frequency-dependency of the RF power absorption for *A*. *aegypti* was investigated by means of numerical simulations at frequencies from 2–240 GHz. This frequency range was chosen to cover both the legacy and 5G telecommunications range and to include wavelengths similar to the mosquito dimensions. To this end, a unique set of high resolution 3D models of yellow fever mosquitoes was developed using micro-computed tomography (CT) scanning. The models were combined with accurate dielectric property measurements in adult yellow fever mosquitoes in order to execute finite-difference time-domain (FDTD) simulations, which resulted in EMF distributions in and around the studied insects. These results provided insights in the power absorption in the mosquitoes’ body, but also in the distribution of this power absorption over the body and different body parts. The novel aspects of this study are (i) the simulations on *A*. *aegypti* in far field EMF exposure between 2–240 GHz, (ii) measurements of dielectric properties in *A*. *aegypti*, (iii) the creation of 3D models based on micro-CT scanning of real mosquitoes, and (iv) assessment of the RF absorption of the full mosquito body and of the different body parts. Our results provide an important input for studies that investigate the spread and biology of *A*. *aegypti* on the one hand and regulators and telecommunication operators who are re-evaluating the guidelines regarding RF-EMF exposure in their planned telecommunication networks on the other hand.

## Materials and methods

The RF power that is absorbed by yellow fever mosquitoes is studied by means of simulations, i.e. solving Maxwell equations numerically. The simulations require a 3D model and dielectric properties of the yellow fever mosquito as inputs. The 3D models were based on scans of real dried yellow fever mosquitoes and the dielectric properties were measured from a mixture of yellow fever mosquitoes.

### Mosquitoes

The yellow fever mosquito specimens were taken from the *Ae*. *aegypti* Rockefeller colony kept in the insectary facility at the Swiss Tropical and Public Health Institute. The mosquitoes were reared at 26–28°C and 60–80% relative humidity with a 12:12 h day:night light cycle. Larvae were fed with ground TetraMin flakes (Tetra GmbH, Melle, Germany) and adults were provided 10% sucrose solution ad libitum. Females were artificially fed with pig blood received from the local abattoir to keep the stock colony, however the specimens used for the measurements in the current study were unfed.

### Scanning and modelling method

In the simulations of RF-EMF exposure of mosquitoes, the models of the insects should be as close to reality as possible. Therefore, real mosquitoes were dried and scanned with a micro-CT scanner to form 3D models. The samples were scanned using the custom-designed HECTOR scanner [26] of the Ghent University Center for X-ray Tomography (UGCT; www.ugct.ugent.be) with a tube voltage of 70 kV and a target power of 10 W. During a full 360°rotation, 2400 projection images were acquired at an exposure time of 1 s each. The samples were positioned on floral foam and positioned close to the X-ray source, resulting in a geometrical magnification of approximately 50. The raw data was reconstructed using Octopus Reconstruction at a reconstructed voxel size of (4.012)^3^ *μ*m^3^. To reduce edge enhancement artefacts and increase the signal-to-noise ratio in the data, the Paganin phase retrieval algorithm was applied [27, 28]. To extract the STL model from the 3D volume, each mosquito datastack was loaded into VGStudio MAX (Volume Graphics, Heidelberg, Germany). Areas where the mosquito touched the floral foam were removed manually. After applying a median filter, the region containing the outer structure of the insect was selected using a growing area function. Although many inside features were not selected with this procedure, leaving large unwanted cavities in the model, this was compensated for by selecting these internal regions separately and merging the volumes. Finally, these regions were converted to a single cohesive STL file per mosquito.

### Dielectric properties

In order to determine the internal EMFs inside the insect models developed in the previous section, Maxwell’s equations can be solved numerically. For this purpose, the dielectric properties, relative permittivity (*ϵ*_*r*_) and conductivity (*σ*) of the insect need to be known and inserted into the equations. The novel dielectric assessment kit for thin layers (DAK-TL) from SPEAG (Schmid & Partner Engineering AG, Switzerland), which is based on the open coaxial probe method, was used to perform dielectric spectroscopy [29]. DAK-TL overcomes the long existing limitation associated with Open-Ended Coaxial Probes (OCP) where sufficiently large samples are needed to avoid reflections at the boundaries. Using full-wave analysis of the open coaxial probe geometry, the complex dielectric properties are calculated from sample thickness and the complex reflection coefficient measured with the vector network analyzer (VNA). The DAK-TL-1.2E probe (5–67 GHz) was used in combination with a ZVA67 VNA (Rohde & Schwarz, Munich, Germany) to perform measurements over the aforementioned frequency range. The measurement resolutions are set to 50 MHz and 250 MHz for the interval 5–6 GHz and 6–67 GHz, respectively. The DAK-TL system was calibrated using the standard 3-point calibration prior to each measurement session: open, short (copper strip), and de-ionized water as the load. A force of 800 N was applied during the short calibration to ensure a good contact between the probe and the copper strip. The validity of calibration was verified by measuring another reference liquid, namely 0.05 M saline solution with known dielectric properties. The measurement uncertainty values (Table 1) that includes possible systematic errors due to design, calibration, thickness measurement uncertainties, and VNA noise were established according to [30].

**Table 1:** Expanded uncertainties (k = 2) in the measurements of dielectric parameters made with the DAK-TL-1.2E Probe.

Frequency (GHz)	Δ*ϵ* (%)	Δ*σ* (%)
5	3.4	4.7
6	3.8	5.0
10	3.4	4.3
15	3.1	3.7
20	3.7	4.1
30	3.6	3.7
40	3.9	3.9
50	4.7	5.0
60	5.1	3.9
67	5.1	4.6

As poikilotherms, the environmental temperature is an important parameter affecting the life of yellow fever mosquitoes. The dielectric characterization was performed at 22°C, this temperature was chosen because it is a temperature where flight activity, host-seeking, and blood-feeding are not impaired. [31] A metallic Petri dish (inner diameter = 30 mm, height = 4 mm) was used to characterize the homogenised mosquito sample. The different tissues of the mosquito are small in sample size (approximatly < 1 mm^3^), dissecting multiple samples is very time consuming and thus it was chosen to work with a carefully prepared homogenized mixture. A reproducible method for preparing an insect-mixture for the measurements was developed. In this procedure, approximately two thousand A. aegypti mosquitoes, including both males and females, were used for dielectric properties measurements. First, the mosquitoes were euthanized prior to measurements using CO_2_. Afterwards, the samples were homogenized using a small battery-operated mortar in combination with the application of mechanical force. This resulted in a homogeneous semi-solid mosquito-mixture of about 2 ml, for which aliquots were prepared.

To measure the complex permittivity $\hat{\epsilon }$ of each of the two samples, the Petri dish filled with the mixture was slowly moved towards the probe and stopped when the distance between the Petri dish bottom plate to the probe was 1±0.02 mm. This was repeated three times for both samples by removing the slurry from the holder and refilling the Petri dish. The complex permittivity of each sample was determined as the average over these three measurements. The complex permittivities used in the simulations are the averaged values from the two sample characterizations.

The studied frequency region of interest extends outside the measured range of 5 to 67 GHz. Therefore, the dielectric properties are extrapolated down to 2 GHz (within the 4G frequency range) and up to 240 GHz (in the millimeter-wave mobile broadband [2]). The properties were extrapolated by the Debye-model [32] whose coefficients were determined from a least mean square fit to the measured complex permittivity of the mixture, as in [24]:

$$\begin{array}{c} \hat{\epsilon }=\epsilon^{\prime }-j\epsilon ”=\epsilon^{\prime }-j\frac{\sigma }{\omega \epsilon_{0}}=\epsilon_{\infty }+\frac{\epsilon_{s}-\epsilon_{\infty }}{1+j\omega \tau }+\frac{\sigma_{s}}{j\omega \epsilon_{0}} \end{array}$$ (1)

with $\hat{\epsilon }$ the complex relative permittivity of the sample, *σ* the conductivity of the sample, *ω* the angular frequency, *ϵ*_*s*_ the static permittivity, *ϵ*_∞_ the permittivity at infinity, *τ* the relaxation time, *ϵ*_0_ the permittivity of free space and *σ*_*s*_ the static conductivity. The measured data is fitted considering firstly one relaxation time and secondly two relaxation times. In the second case, Eq 1 for the real and imaginary part (loss factor) of the relative permittivity reads as:

$$\begin{array}{c} \epsilon^{\prime }=\epsilon_{\infty }+\frac{\Delta \epsilon_{1}}{1+j\omega \tau_{1}}+\frac{\Delta \epsilon_{2}}{1+j\omega \tau_{2}} \end{array}$$ (2)

$$\begin{array}{c} \epsilon ”=\frac{\sigma }{\omega \epsilon_{0}}=\frac{\sigma_{s}}{\omega \epsilon_{0}}+\frac{\Delta \epsilon_{1}\omega \tau_{1}}{1+(\omega \tau_{1})}+\frac{\Delta \epsilon_{2}\omega \tau_{2}}{1+(\omega \tau_{2})} \end{array}$$ (3)

where Δ*ϵ*_*i*_ is the difference between the static permittivities *ϵ*_*s*,*i*_ and the permittivity at infinity *ϵ*_inf_, with *i* = 1, 2 corresponding to the two relaxations.

### Numerical simulations

**Fig 1:**
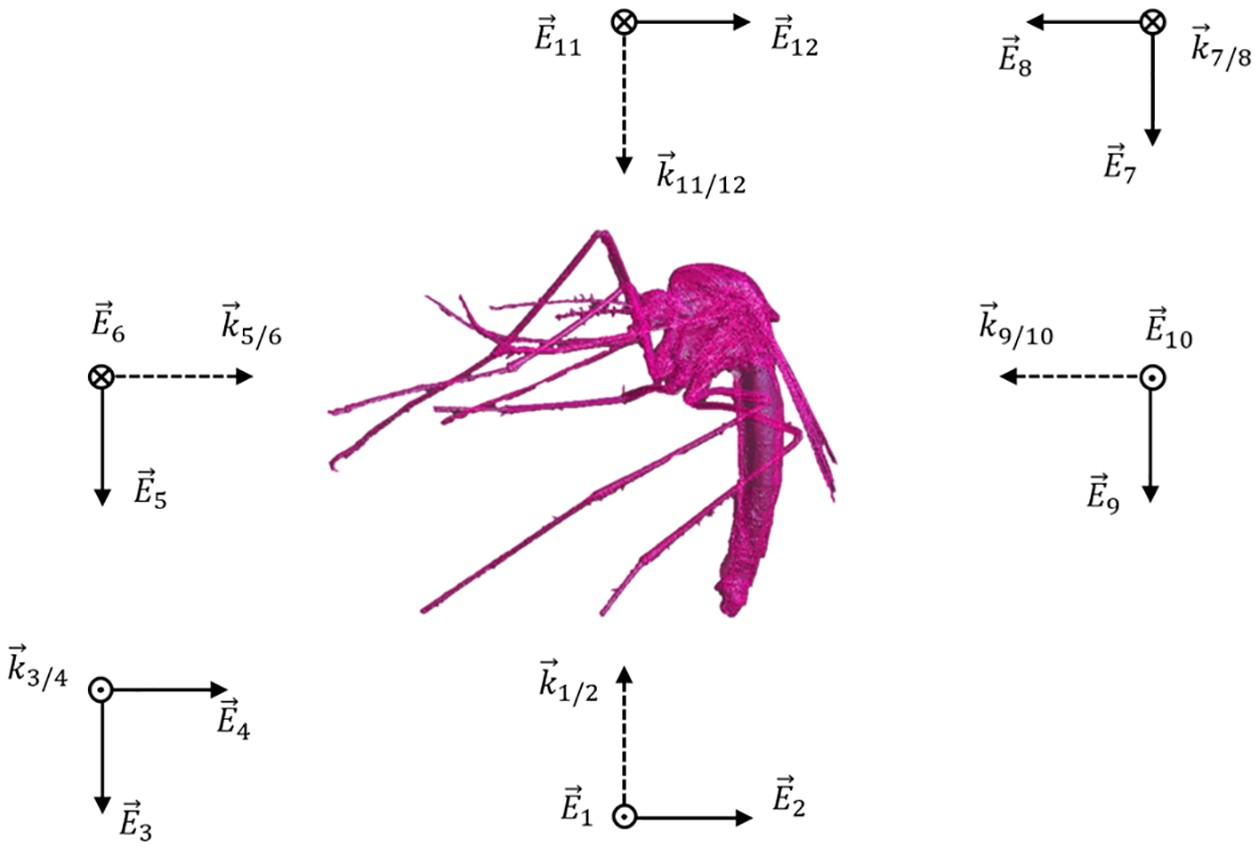
An overview of 12 plane waves in the simulation set up. 6 directions × 2 polarizations. The wave vector is indicated with the dotted arrow and the electric field with a solid arrow.

The mosquito models and the measured dielectric properties were used in numerical simulations with the commercial software Sim4Life Version 5.2.1 (ZMT Zürich MedTech AG, Zürich, Switzerland), where the FDTD method was used to determine the internal electric field inside and around the mosquito body. This study investigated exposure in the far-field or Fraunhofer radiation region. In this zone the following conditions hold for the separation distance between insect and RF-EMF source (*r*): *r* ≫ 2*l*^2^/λ with λ the wavelength and *l* the largest dimension of the RF source and the insect [33]. In this region, any RF-EMF field can be described as a set of incident plane waves [33]. This technique was previously used to determine the RF-EMF absorption in heterogeneous human body models [3] and for other insects [17, 18]. The dielectric heating is proportional to the the total absorbed RF-EMF power (*P*_*abs*_), which in its turn can be found from the internal electric field:

$$\begin{array}{c} P_{abs}=\int \sigma \times {\left|{\vec{E}}_{int}\right|}^{2}\cdot dV \end{array}$$ (4)

with *σ* the conductivity, $\left|{\vec{E}}_{int}\right|$ the root mean square internal electric field strength and *V* the volume of the insect. *P*_*abs*_ depends on the frequency, as both *σ* and *E*_*int*_ are dependent on the frequency. Therefore, different frequencies were investigated: 2, 6, 12, 24, 60, 90, 120 and 240 GHz. For a flying or resting mosquito, the polarization and angle of incidence of far-field EMFs is unknown a priori. Therefore, twelve incident plane waves were considered to model the far-field exposure, as shown in Fig 1. This was the same configuration as used in [3]: 6 directions along Cartesian axes with 2 orthogonal polarizations per direction. The insects were aligned with the length of the body along one of the axes. In the simulations, all plane waves had a root mean squared electric field strength of 1 V/m. In real situations, this field strength can vary from 1 V/m, since the absorbed RF power scales quadratically with the incident field strength, it can be calculated for an arbitrary field strength given the value at 1 V/m:

$$\begin{array}{c} P_{abs,real}=P_{abs}\left(1V/m\right)\cdot \frac{E_{real}^{2}}{{\left(1/m\right)}^{2}} \end{array}$$ (5)

with *E*_*real*_ and *P*_*abs*,*real*_, the incident electric field strength and absorbed power under realistic exposure conditions.

FDTD is a time-domain method, which has to be terminated after a certain simulation time, which can be quantified in number of periods of the RF-EM waves that is incident on the insect. After a certain number of simulation periods of the incident plane wave, *P*_*abs*_ converges to a steady state. The number of periods required to reach the steady state depends on the size of the simulation domain relative to the simulated wavelength. For a smaller wavelength, more periods will be needed to cover the complete domain. The number of periods were 7 and 35 at 2 and 240 GHz, respectively, while for the other frequencies the number of periods was between 10 and 35. The number of periods was always higher than twice the maximum body length of the mosquito, divided by the wavelength.

In the FDTD method, a grid is imposed to the simulation domain discretizing the volume of interest, including the mosquito. The grid step size was defined according to a trade off between a shorter simulation time for large grid step sizes and a better spatial resolution for smaller grid step sizes. Further, the grid step size should be minimum 1/10 of the wavelength. [34]. The smallest wavelength $\lambda /\sqrt{\epsilon_{r}}$ is 573.5 *μm* at 240 GHz, while all mosquitoes were discretized in voxels of 25 *μ*m.

The FDTD technique used in this paper also has its limitations. The method is based on the discretization of the differential form of Maxwell’s equations [34]. The evolution over time of EMFs is discretized in temporal steps and the simulated space is discretized into voxels. The smaller the voxels and time intervals, the more realistic the simulations are. However, simulations are always an approximation of reality. The simulation domain cannot be infinitely large, and consequently boundary conditions are used to limit the domain. The boundary conditions used in our simulations are Uniaxial Perfectly Matched Layers, which mimic an infinitely extended free space. The simulations further rely on accurate dielectric properties and 3D models.

## Results

### 3D models

**Fig 2:**
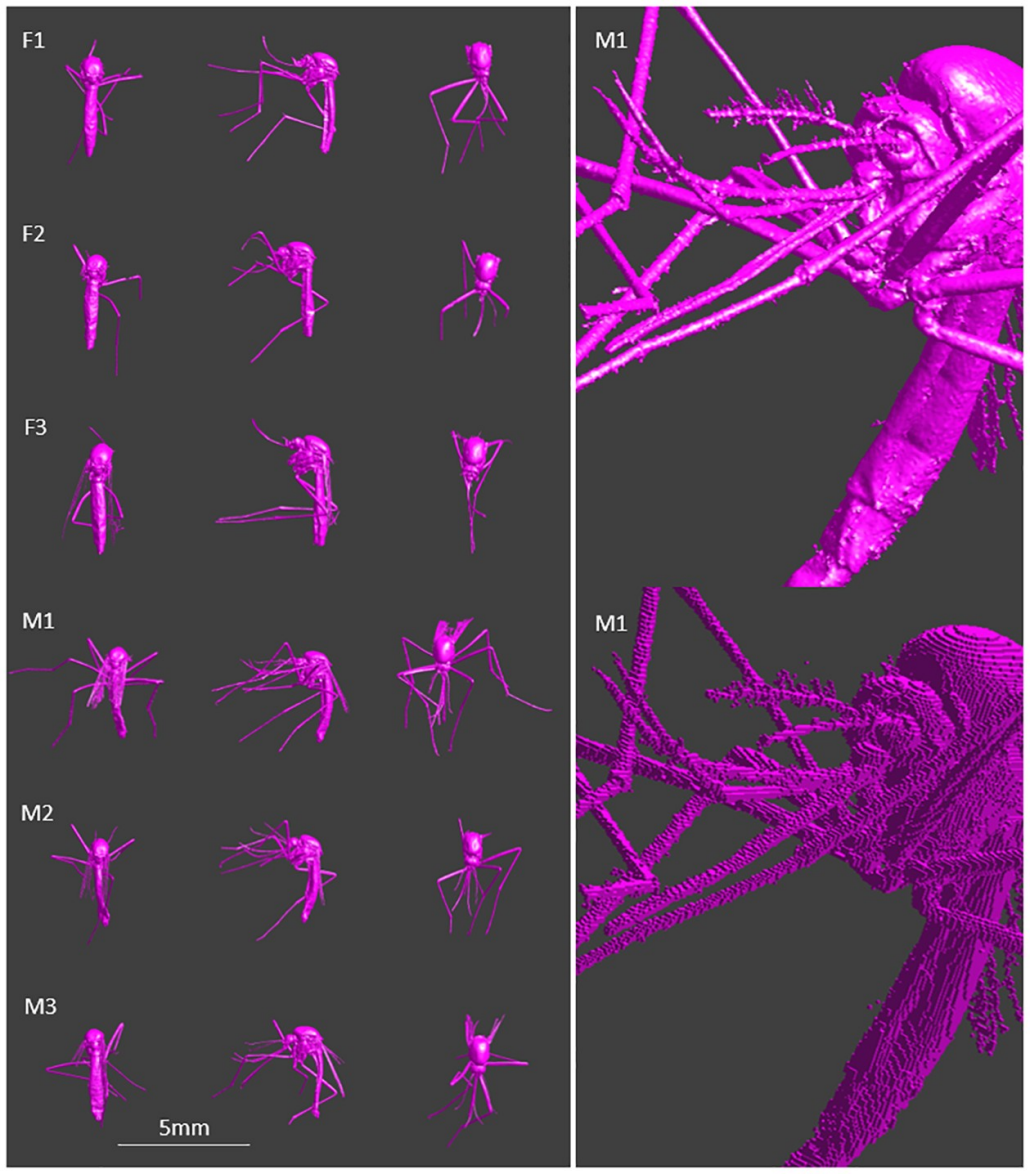
3D models of mosquitoes. Left: Overview of models used in simulations (F: females, M: males). Left to right: Back, side and top view. Right: Mosquito M1 as triangulated model (top) and voxelated model (bottom) with Voxel size 25 *μ*m.

Three dimensional numerical models of three female and three male mosquitoes were constructed from micro-CT scans of real mosquitoes. Using the micro-CT technique, internal structures of the insect under investigation could be distinguished, and a high resolution is achieved. However, fine structures such as parts of the wings, scales or other fine structures, were not distinguishable enough from the surrounding air. Hence, they could not be included in the model. Small details on the thorax, abdomen, and head were manually added on slices based on unsegmented features in the raw data. On the abdomen, many fine structures were not captured, especially for the male models, and were impossible to reconstruct completely. During handling, also loss of legs occurred for some samples. Fig 2 shows the top, side and back view of all six mosquitoes as well as a more detailed view of mosquito M1 for the triangulated model and the model after voxelating (voxel length 25 *μ*m).

Table 2 lists an overview of the volumes and dimensions of the mosquito models. The volumes are taken after voxelating with a voxel length of 25*μ*m. The body length of the mosquitoes is measured from the pronotum to the end of the abdomen. The diagonal given in the table, is the diagonal of the bounding box containing the whole insect. This diagonal strongly depends on the presence of legs in the model, therefore the body length is the main representation of the mosquito dimensions in this paper.

**Table 2:** Volumes and lengths of mosquitoes.

Insect	Volume (mm^3^)	Body length (mm)	Diagonal (mm)
F1	1.103	3.985	7.792
F2	1.083	3.774	4.495
F3	1.402	4.287	7.418
M1	0.913	3.586	9.226
M2	0.691	3.417	7.437
M3	0.833	3.629	7.748

### Dielectric properties

**Fig 3:**
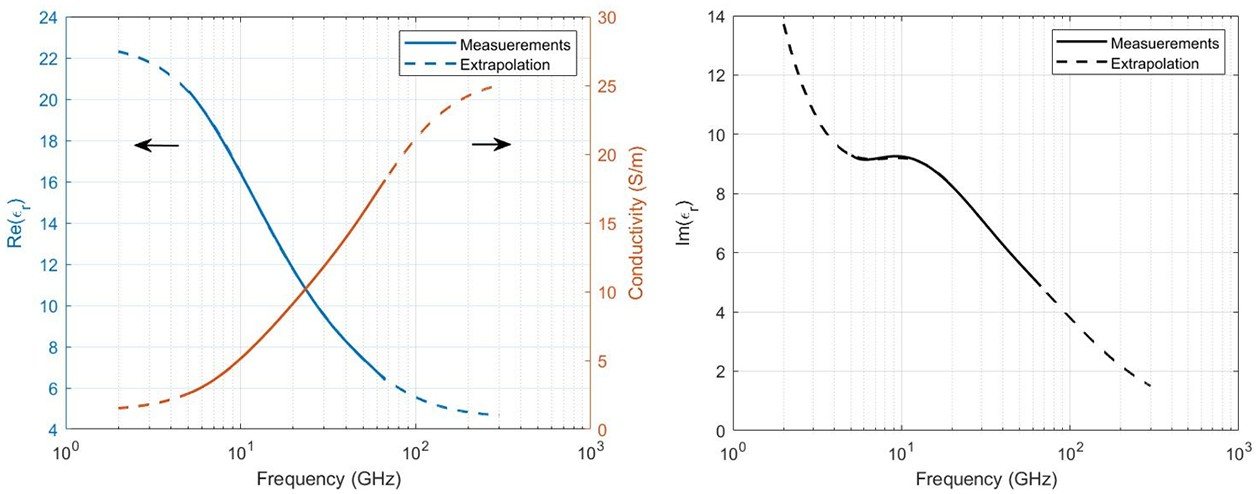
Dielectric parameters. Left: Measured (solid line) real part of the relative permittivity (blue) and conductivity (orange) and the extrapolation (dotted line). Right: Measured (solid line) imaginary part of the relative permittivity and the extrapolation (dotted line).

The insect are approximated as homogeneous models in the simulations, with no differentiation between different tissue and so only one set of dielectric properties are used for the material for every frequency. Fig 3 shows the measured permittivity and conductivity of the mosquito-mixture for frequencies between 5–67 GHz, and extrapolated to 2–300 GHz. The loss factor (imaginary part of the complex permittivity) was found from the conductivity (Eq 2) and is also given in Fig 3. The extrapolation was done using the Debye relaxation model with two characteristic relaxation times, the resulting parameters for the dielectric curves are given in Table 3. The R^2^ for the Debye fit to the measured data is 0.99995 for the real part of the permittivitty and 0.999994 for the conductivity with two relaxations, compared to 0.996 and 0.992 respectively for one relaxation time.

**Table 3:** Parameters for Debye curves, with two (fit 2) and with one (fit 1) relaxation.

	*τ*_1_ (*ps*)	Δ*ϵ*_1_	*τ*_2_ (*ps*)	Δ*ϵ*_2_	*ϵ*_∞_	*σ*_*s*_ (*S*/*m*)
*ϵ*’ fit 2	3.493	4.978	14.68	13.24	4.544	
*σ* fit 2	2.477	4.213	12.23	12.89		1.297

The real part of the permittivity decreases with increasing frequency and the conductivity increases with increasing frequency. The loss factor has a local maximum around the frequency corresponding to *τ*_2_, the most prominent relaxation in this frequency range. At lower frequencies a decreasing trend with increasing frequencies is apparent, originating from the static conduction term *σ*_*s*_/*ωϵ*_0_.

### Simulations

**Fig 4:**
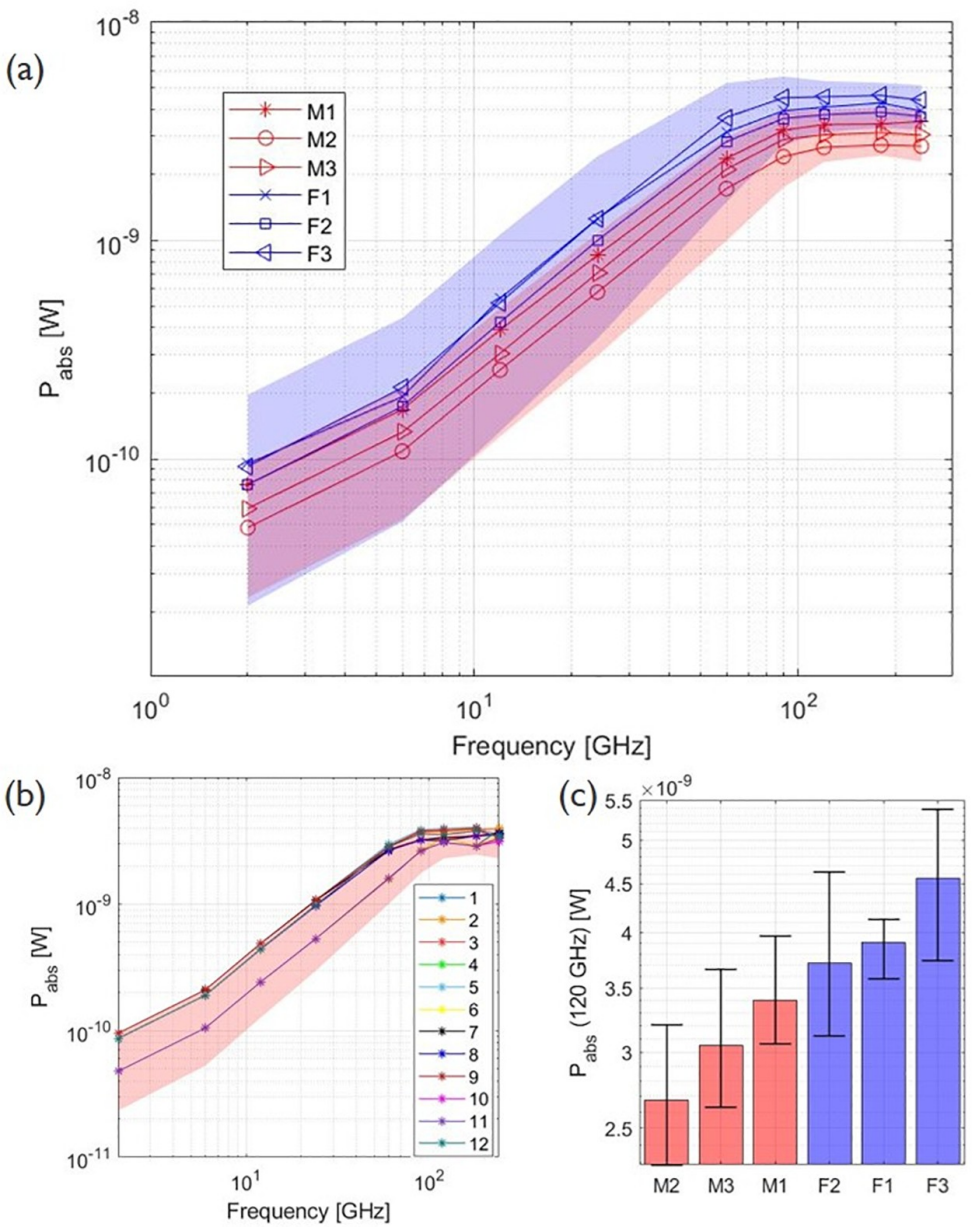
Total absorbed RF power (*P*_*abs*_) by the mosquito as a function of frequency for an incident field strength of 1 V/m. (a) The dots present the means of the 12 plane waves, the blue and red shaded regions represent the range of absorbed power for all 12 plane waves for all female and all male mosquitoes respectively. (b) The absorbed powers for mosquito M1 for the twelve different plane waves as illustrated in Fig 1. The red shaded area represents the range of absorbed power for all 12 plane waves for all male mosquitoes. (c) The bar chart shows the total absorbed power (*P*_*abs*_) at 120 GHz, the whiskers indicate the range for all 12 plane waves.

The amount of RF power absorbed by the 3 male and 3 female mosquito bodies in the far field is shown in Fig 4 as a function of frequency. The 3 blue and 3 red lines show the means of *P*_*abs*_ for the 12 configurations (Fig 1) in which the the 3 female and 3 male mosquitoes were exposed, respectively. The shaded areas in the graph include all 12 *P*_*abs*_ values for all 3 females (blue) and 3 males (red) of the simulations. *P*_*abs*_ increases with increasing frequency, up to 90 GHz for all six mosquitoes. At 90 GHz the highest single plane wave absorbed a power of 5.64 nW and was found for the F3 model with plane wave configuration 5 (Fig 1). At this frequency, the wavelength of the incident plane wave becomes comparable to the body length of the mosquitoes. Between 120 GHz and 180 GHz, the averages of *P*_*abs*_ for all mosquito reach a maximum, here the wavelength is smaller (2.5 mm and 1.7 mm respectively) than the body length of the insect but still comparable to the insect dimensions. Fig 4B shows *P*_*abs*_ for the 12 plane wave configurations for mosquito M1 and Fig 4C shows the average *P*_*abs*_ for all mosquitoes at 120 GHz.

**Fig 5:**
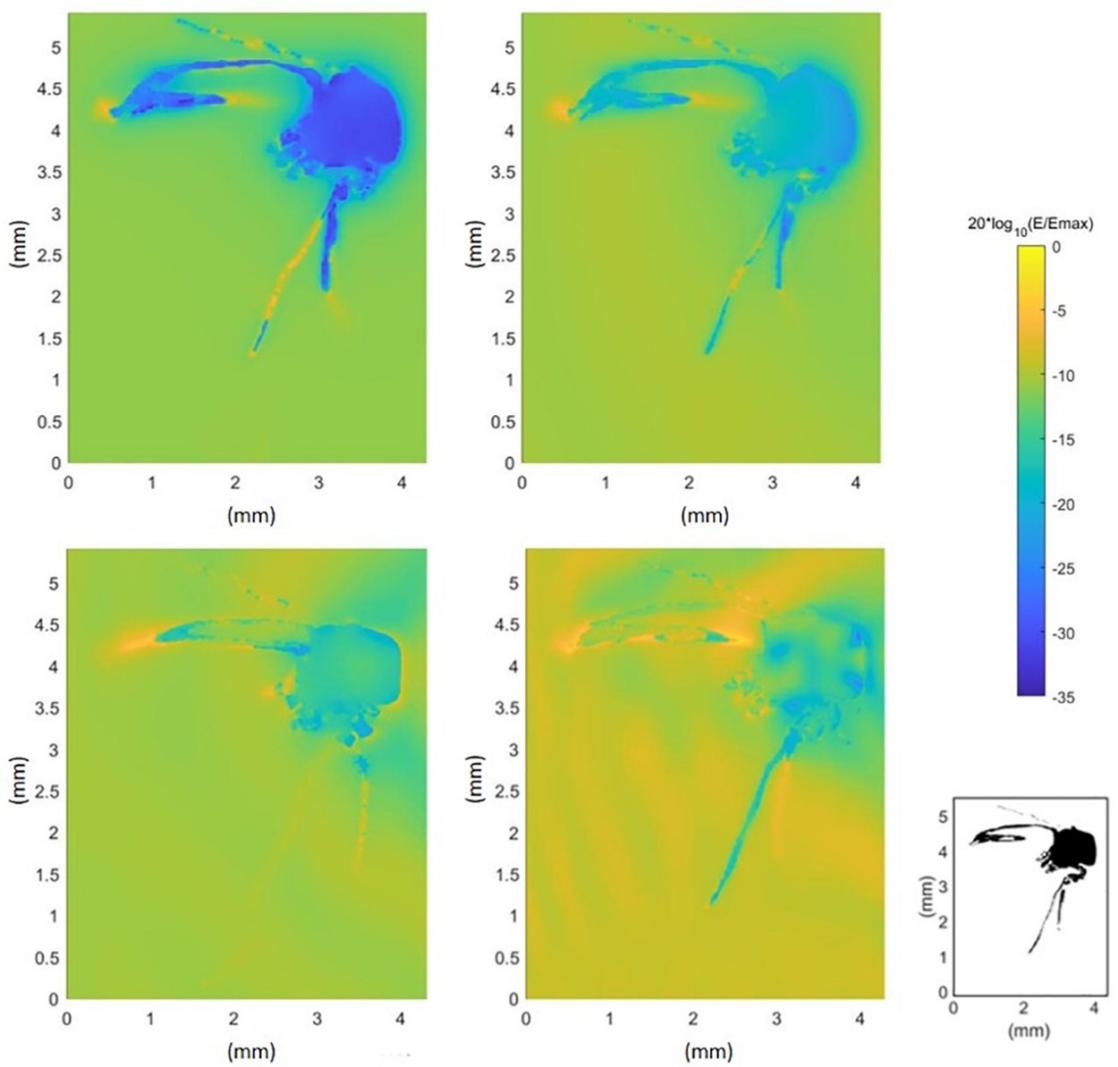
Normalized electric field strength in and around a mosquito. Electric field strength (dB) normalized to the maximal electric field strength of the simulation (*E*_*max*_) in the cross sections of mosquito M1 at 6 GHz (top left), 60 GHz (top right), 120 GHz (bottom left) and 240 GHz (bottom right). Configuration 1 (Fig 1) was used for all simulations. The bottom right panel shows a cross section of the mosquito.

From Eq 4, it is clear that the power absorbed by the insect depends on the internal electric field strength. Fig 5 shows the normalized electric field strength (dB) in and around mosquito M1, presented in mid-sagittal cross sections. The normalization was done to the maximum electric field strength of every simulation separately. The maximal and minimal electric field strength found in the 6 GHz simulation in exposure configuration 1, was 3.53 V/m and 1.29 × 10^−4^ V/m respectively. For 240 GHz, this was 2.72 V/m and 2.29 × 10^−2^ V/m respectively. It should be noted that the abdomen does not contain a large cavity, it is the dried abdomen that is curved.

**Fig 6:**
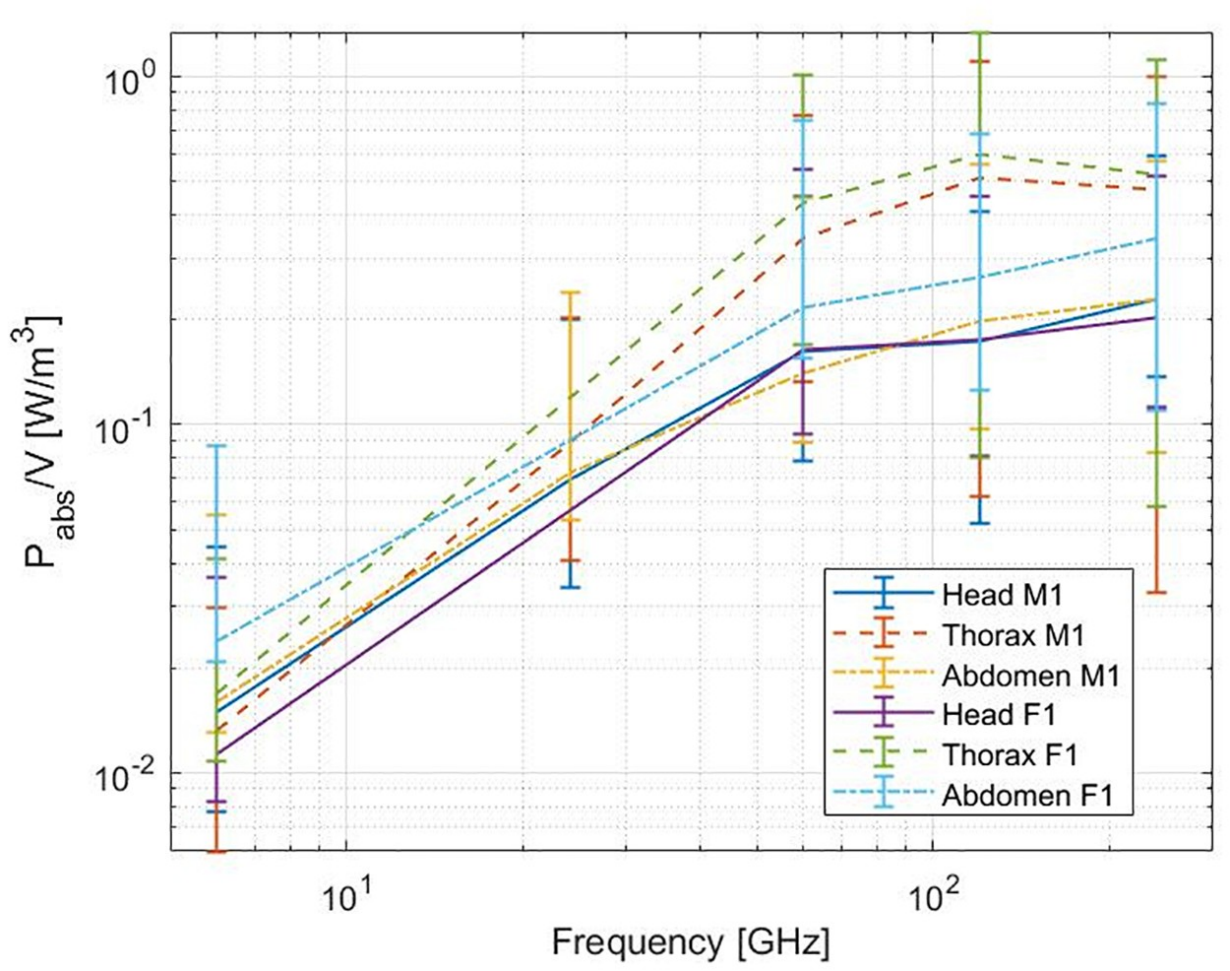
Averaged absorbed power as function of frequency in the head, thorax, and abdomen of a yellow fever mosquito For an incident field strength of 1 V/m. Averaged over the volumes of the body part for mosquitoes M1 and F1.

In studies that investigate absorption of RF-EMF fields, a differentiation between absorption in different body parts is often made. In the case of humans, for example, the international committee on non-ionizing radiation differentiates between RF-EMF exposure of the torso and head on the one hand and the limbs on the other hand [35]. This approach is followed because exposure or heating of different body parts might results in different outcomes. Using the simulations performed in this study, the different parts of the mosquito body can be considered separately as well. The absorbed power in the head, thorax, and abdomen were calculated and averaged over their respective volumes. The volumes selected as the body part varied slightly for simulations at different frequencies due to different grid settings, and thus the volumes varied slightly as well. The volume of the head, thorax, and abdomen were 0.91 ± 0.01 mm^3^, 4.30 ± 0.03 mm^3^, and 2.91 ± 0.05 mm^3^ for M1, respectively. The average *P*_*abs*_ for M1 and F1 are given in Fig 6. The head and abdomen have a similar behaviour, while there is more averaged *P*_*abs*_ at higher frequencies for the thorax. For the female mosquito, the thorax and abdomen averaged *P*_*abs*_ are slightly higher.

#### Effect of model variations

The *P*_*abs*_ is slightly larger for the three females than for the three males (KS-test, p<0.05). This can partly be explained by the larger volume and body size of the females [36] as seen in Fig 2.

In addition to the sex-related differences, the models varied in number of legs still attached to the specimens. Model M1 had 6 legs, while F3 had only two. The influence of the presence of the legs is investigated by removing a hind leg of M1 and by removing all six legs of M1. Simulations were again done at 120 GHz for the 12 different plane waves and compared to the results for the original six-legged M1, which had a mean *P*_*abs*_ of 3.40 nW for the 12 plane waves at 120 GHz. When the hind leg of M1 was removed, the volume of the mosquito model was reduced to 97.4%, and the *P*_*abs*_ mean decreased to 3.31 nW with a maximal relative deviation of 3.39% for the 12 simulations. For the case where all legs were removed, the model volume was reduced to 86.0% of the original model and the mean of *P*_*abs*_ decreased to 2.91 nW, with a maximal relative deviation of 20.92% for the 12 simulations.

#### Uncertainties of simulation

It becomes clear from Fig 4 that the angle of incidence and polarization of the plane waves, have an impact on the absorbed power. The 12 plane waves seen in Fig 1 are orientated along the main axis of the insects. Real life exposure situations are not limited to those 12 configurations and simulations with 30 random orientations and polarizations were added at 60 GHz for M1. None of these simulations resulted in a *P*_*abs*_ outside the interval of the 12 plane waves along the main axes. The mean of *P*_*abs*_ for the 30 simulations was 2.31 ± 0.36 nW, which is comparable to the mean of *P*_*abs*_ for the 12 plane waves 2.37 ± 0.59 nW, for *E*_*inc*_ = 1 V/m.

Another element contributing to the uncertainty in the simulations is the grid step size or voxel size. The voxel size of the mosquito models was set to 25 *μ*m, and is larger than the resolution of the mosquito models. To confirm the chosen grid step size is small enough, 12 simulations (six incident angles × 2 polarizations) with a smaller grid step size of 15 *μ*m were performed at 240 GHz. At this frequency, the wavelength is the smallest and the voxel size becomes more important. Compared to the case with 25 *μ*m, *P*_*abs*_ for the 15 *μ*m has a maximal relative deviation of 2.61% and has a mean *P*_*abs*_ of 3.49 nW (versus 3.51 nW). This smaller voxel size has a smaller impact on *P*_*abs*_ than the incident angle and polarization, hence the grid step size of 25 *μ*m can be considered sufficient for these simulations.

The measurement of the dielectric properties was done for two samples of a mosquito-mixture, with a maximal relative deviation between the two samples on the dielectric properties of 4.04% and 5.81% for *ϵ*′ and *σ* respectively. To establish the influence of the dielectric parameters on the simulation results, four extra sets of 12 simulations were executed for a plane wave of 60 GHz (*ϵ*′ ± 0.0404 × *ϵ*′, *σ* ± 0.0581 × *σ*). A maximal relative deviation on *P*_*abs*_ was found to be 5.41%, which again is smaller than the influence of incident angle and polarization.

## Discussion

### 3D Models

We used micro-CT scanning to obtain spatially accurate mosquito models that had a sufficient resolution to model exposure at 240 GHz (grid step = 25 *μ*m). The micro-CT approach has the advantage of being non-invasive and providing information of the insect’s internal anatomy [37]. However, dried specimens were used for the micro-CT scans to reconstruct the models in this study and the models were made homogeneous with no distinction between different tissues in the mosquito. Real mosquitoes will have different tissues and the position, size, gradients and edges of these tissues will influence the power absorption. In [17, 18] a similar technique was used for different life stages of the Western Honeybee, an Australian Stingless Bee, a Desert Locust and a Dor Beetle.

Other possible techniques for imaging of insects exist [38, 39], but are not all suitable for reconstruction of 3D models. An example of 3D reconstructions of insects is given in [40], where a camera and different angles and focal depths were used with shape-from-silhouette. In [41], a shape-from-motion approach was used for capturing insects’ surface geometries and colours. They were able to capture details such as hairs for different insects and a 3D mosquito model from a *Culex pipiens L*. was retrieved using this method. However the resolution of both techniques was lower than for the 3D mosquito models used in this study. In [42] 3D models of insects constructed by a structured light scanner also showed less details of the surface geometries than for a (Synchrotron Radiation) micro-CT method. To the best of our knowledge, no other 3D model of a yellow fever mosquito exists that has been constructed from data of a real insect. Moreover, our mosquito models are based on micro-CT scannings that have a high resolution in comparison to most existing models.

However, the micro-CT method has its limitations: using this imaging technique, some details such as scales and thin antennae were not distinguishable from air in the 2D cross-sections and are thus not part of the 3D model. Additionally, the scanned insects need to be immobilized or dead. In the case of dried species, small deformations can occur compared to living mosquitoes. Nevertheless, the 3D models based on mosquito specimens are detailed and can be considered a good representation of life mosquitoes. Our models can be used to obtain realistic values of absorbed power and inspection of actual *E*_*int*_ distributions in simulations.

### Dielectric properties

**Fig 7:**
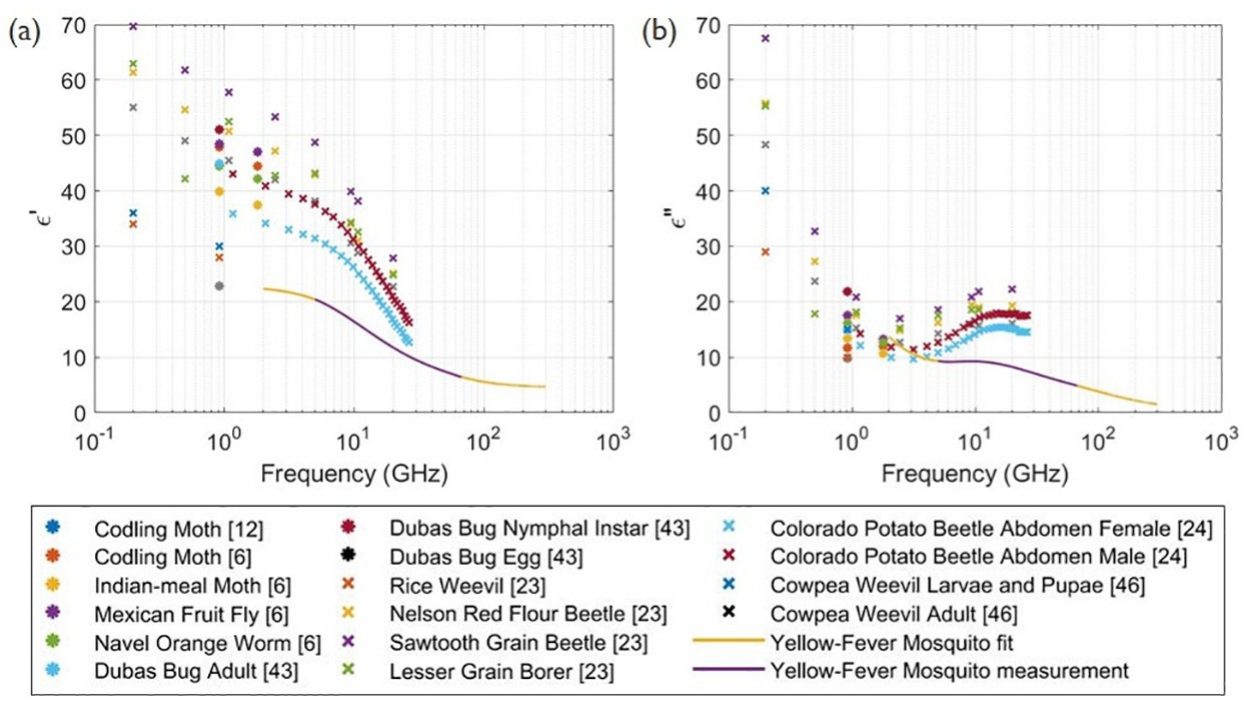
(a) Real and (b) imaginary part of relative permittivity. Given for insects in other studies [6, 12, 23, 24, 43, 45, 46] and for the yellow fever mosquito.

Dielectric properties of the yellow fever mosquito mixture were obtained using coaxial-probe measurements from 5–67 GHz. Previous studies [6, 23, 24, 43–46] have also investigated dielectric properties of insects in this frequency range, some of these values for *ϵ*′ and *ϵ*” are given in Fig 7, together with the dielectric properties from this study for comparison.

The dielectric properties are slightly smaller for the yellow fever mosquito than most of the other insects measured in [6, 23, 24, 45, 46] for the same frequency range, but are overall comparable, see Fig 7. The permittivity curves exhibit similar behaviour to each other and to the Debye model, with *ϵ*′ decreasing with increasing frequency. From 6 GHz up to higher frequencies, the *ϵ*” shows a typical Debye relaxation response, for *τ*_2_ a local maximum is visible in Fig 3 around 9.4 GHz. This same behaviour was also observed in previous studies on insect dielectric properties: a local peak is also found around 9.4 GHz for the red flour beetle and the lesser grain borer in [23], around 15.81 GHz for the Colorado potato beetle in [24] and between 10–15 GHz for the larva of the palm weevil in [44]. At lower frequencies, a decreasing trend with increasing frequencies is apparent for our model (Fig 3) and the measurements presented in [23, 24, 46], originating from the static conduction term.

When two relaxation times are assumed in the Debye fit of the the dielectric properties, the curves *ϵ*′ and *ϵ*” are well in agreement with the measurements, better than for the case of only one relaxation. The parameters of the Debye model given in Table 3, are similar for both curves, a confirmation that the two relaxation Debye model is suitable. For simulations with frequencies below 5 GHz or above 67 GHz, the dielectric properties are taken from a extrapolation using these parameters.

### Numerical simulations

The absorption of RF power by the mosquito is dependent on the electric field in and around the insect, described by Eq 4. At 6 GHz, the wavelength of the incident wave is considerably larger than the insect and will mostly refract and not penetrate the insects’ body. At this frequency, it can be seen in Fig 5 that the electric field strength is higher at the boundaries of the insect than inside, making the surface area of the insect an important factor in the power absorption. For increasing frequency up to 120 GHz, EMFs penetrate the insect more efficiently, contributing to an increasing absorption. This is visible in Fig 4. At a frequency of 90 GHz, the increase in absorbed power becomes smaller and the maximum value of *P*_*abs*_ is found for mosquito F3 in configuration 5 (Fig 1) with the electric field parallel to the body length of the insect. The incident wave with a frequency of 90 GHz corresponds to a wavelength of 3.33 mm (in free space), comparable to the body length of the insects (Table 2). In this frequency region, whole-body or partial-body resonance [3] occurs causing a higher absorption of the EMFs. The electric field will penetrate more in the insect (Fig 5) at this higher frequency compared to a lower frequency, 6 GHz, inducing more RF absorption inside the insect (Eq 4). On the contrary at 240 GHz, the penetration depth is expected to decrease [18] in comparison to 90 GHz, and spots with lower electric field strength are found in the (middle of the) thorax. Simultaneously, the conductivity increases with increasing frequency as shown in Fig 3. The combination of these two counteracting effects cause the *P*_*abs*_ to slightly decrease at 240 GHz in comparison to 90 GHz. At frequencies below 90 GHz, incident electric fields oriented along the same axis induce a *P*_*abs*_ in the insect, which are nearly equal to each other. This can be seen in Fig 4B where the *P*_*abs*_ under exposure with the electric field along the three main axes show three bundles of four coinciding values at these frequencies below 90 GHz. The relative order of *P*_*abs*_ induced by the polarization below 90 GHz is altered for higher frequencies and the polarization responsible for lowest or highest *P*_*abs*_ varies.

At 6, 60 and 120 GHz, the mean *P*_*abs*_ for an incident field strength of 1 V/m, of all mosquitoes and all 12 plane waves, are 0.165 nW, 2.64 nW and 3.59 nW respectively. For a change of 6 GHz to 60 GHz with the same incident field strength (1 V/m), this translates into a power absorption that is 16 times higher. For a change from 6 GHz to 120 GHz this increase will be even greater, the *P*_*abs*_ is 21.8 times higher. In the current networks, frequencies up to 6 GHz are used, with most telecommunication frequencies at ≤ 2 GHz [1]. Future networks that emit EMFs at higher frequencies with a same incident power, will consequently lead to more absorbed power by yellow fever mosquitoes. In reality, the incident electric field strength will vary in time and position. Currently, 5G networks have started being installed and the typical values of the electric field strengths are not yet known for all situations. From measurements at 3.5 GHz in [47] a maximal electric field strength of 4.9 V/m was found at their measurement location, after scaling to a input power of 200 W on the base station. In simulations designed to decrease exposure in a 5G networks presented in [48] at 3.7 GHz, the authors of [48] expected electric field strengths between 0.0068 and 0.0233 V/m in a crowded environment. The values can thus be lower or higher than the 1 V/m that was used in the simulations with the mosquitoes (see Fig 4). Additionally, the exposure in the environment will be limited by (inter)national guidelines and legislation. Many of these are based on the ICNIRP guidelines [35]. The ICNIRP reference level is 61.5 V/m at 2 GHz for the general public when averaged over 30 minutes, which is 61.5 times larger than the electric field strength used in the simulations in this manuscript. For larger frequencies, up to 300 GHz, the guidelines specify a limit on the incident power density, which is 10 W/m2, instead of the incident electric field strength. The absorbed powers in this work can be rescaled to other incident electric field strengths using Eq 5. Further, dielectric heating will be caused by exposure at multiple frequencies simultaneously. Absorbed RF power is a proxy for dielectric heating, which can have an effect on e.g. behaviour, development and dielectric heating of mosquitoes might influence their spread. In order to assess dielectric heating accurately, more precise measurements of A. Aegypti’s mass and specific heat capacity would be necessary.

In [17], similar simulations were performed on four insects which resulted in a similar power absorption dependency on frequency. The largest absorption occurred at frequencies with a wavelength comparable to the insect’s size. The smallest insect under consideration, the Australian Stingless Bee, experienced less power absorption compared to the larger insects for all frequencies, with a maximal *P*_*abs*_ at 60 GHz of ≈ 30 nW. The body lengths of the Australian Stingless Bee models are comparable to the length of the mosquitoes, however the peak of maximal absorption is at a lower frequency and *P*_*abs*_ is higher than for the mosquitoes. This indicates the necessity of insect-specific simulations and measurements of dielectric properties. Going from 6–60 GHz (the absorption peak), meant for the Australian Stingless a higher *P*_*abs*_ by a factor ≈ 23. For the mosquitoes, this difference (×16) is smaller, however when looking at 6–120 GHz, i.e. looking at an increase from 6 GHz up to the plateau around the maximum *P*_*abs*_, a similar increase of a factor ×21.8 is seen. For the other insects in [17], the absorption peak is found at even lower frequencies (6, 12 and 24 GHz). In [18], different life stages of the Western Honeybee were subjected to similar simulations, also here a similar power absorption dependency on frequency was observed and absorbed powers were again higher than for the mosquitoes. Going from 6 GHz to higher frequencies in [17, 18], meant going up in absorbed power by the insect, except for the Honey bees that had an absorption peak at 6 GHz.

The power absorption is not proportionally distributed over the body, and thus not only the whole-body absorbed power is considered, the mosquito body is divided in three parts without the legs and the absorbed power is averaged over this body part. Fig 6 shows the averaged head, thorax and abdomen *P*_*abs*_ for M1 and F1. This gives insight in the absorption in the different parts of the insect. Abdomen and head have a similar average absorption, and the thorax has a considerably larger averaged absorption at 60 GHz and higher. The thorax has a clearly higher volume-averaged RF absorption than the head or abdomen. We attribute this difference to the shape, the mosquito models have a thorax that can be approximated as a solid sphere, while the abdomen and head have a larger surface/volume ratio than the thorax.

#### Effect of model

From Fig 4, a larger *P*_*abs*_ is found for the three female mosquitoes for all frequencies, than for the three male mosquitoes. When comparing the volume of the mosquitoes in Table 2, it is clear that the female mosquitoes under consideration, have a larger volume and thus can absorb more RF power. When averaging the *P*_*abs*_ over the volume of the whole body, the three female mosquitoes still show a higher RF absorption than the three males. The radar cross section does not scale linearly with volume. The female mosquito is not only larger, but differs also slightly in morphology from the male mosquito [36], which also induces a difference in *P*_*abs*_. Further, the models used in the simulations, do not all have the same amount of legs, which influences the absorbed power. Absorbed power in models with less than 6 legs, would have a higher absorbed power in the realistic case of six legs.

#### Uncertainties of simulations

The FDTD technique makes use of a three dimensional grid and the mosquito models need to be discretized. The voxels used in all simulations were 25 *μ*m. Smaller voxels result in more reliable results, however simulation times will run up. The influence of a smaller voxel size and the influence of the uncertainty on dielectric parameters are smaller than the effect of incidence angle and polarization. It follows that a choice of 25 *μ*m for the voxels is sufficient. The impact of incidence angle and polarization on the *P*_*abs*_ is visible from the bar chart in Fig 4, where the whiskers indicate the total range of *P*_*abs*_ for the 12 simulations. To verify that these 12 plane waves represent the range of possible absorbed powers, 30 simulations with random orientations and polarizations were considered at 60 GHz. No simulation resulted in a value outside the range of the earlier 12 plane waves.

### Strengths and limitations

This paper contributes to the state of the art in different aspects. First of all, six detailed 3D models of the yellow fever mosquito were designed by means of micro-CT scans. The models are, to our knowledge, the first models based on real mosquitoes with a high spatial resolution. Second, the dielectric properties of homogenised yellow fever mosquitoes were measured for the first time, in a 5–67 GHz range. The use of the model obtained from real mosquitoes and the use of dielectric properties of the insect in question, result in more accurate simulations. Advantages of using simulations as opposed to experiments, is the ease in exploring different setups and frequencies. The RF exposure of mosquitoes were numerically simulated, leading to first-time insights on absorbed power by yellow fever mosquitoes at frequencies in 4G and the future 5G mm-waves.

The methods used in this study also come with limitations. The micro-CT scan, despite leading to high resolutions and insight in internal structures of the insects, did not capture the wings and certain thin instances of the mosquitoes. The models were made from dried unfed dead mosquitoes, and no distinction was made between different tissues of the insect. Real mosquitoes will have different tissues and the position, size, gradients and edges of these tissues will influence the power absorption. The dried species can vary slightly from living mosquitoes in form. The dielectric properties below 5 GHz and above 67 GHz, are not measured but acquired by extrapolation. In the simulation environment, the model is discretized in voxels and the simulations are only an approximation of reality. The limitations of the simulations further lie in the uncertainties that accompany the FDTD technique and the use of a limited amount of plane waves representing the far-field. Uncertainties on parameters in simulation settings and random angle of incidence were adopted in extra simulations to explore these limitations. However the absorbed power is a proxy for dielectric heating, values of actual heating are outside the scope of this paper.

### Future work

Future work will consist of the study of other life stages of the mosquito exposed to RF-EMF exposure. Another step forward will be investigating heterogeneous models of the insect, with different dielectric properties for different tissues. Experiments concerning RF-EMF absorption, scattering and measurements of heating (by e.g. infrared cameras), can verify current results and give more insight in the matter. Also measurements on living mosquitoes will enable us to study the influence of RF EMFs on the insect. Further, other insects can be used in similar simulations, to have a more complete view of RF-EMF absorption of insects exposed to 4G and 5G telecommunication and size dependency of absorbed power.

## Conclusion

By creating six high resolution 3D models and by measuring dielectric properties from real mosquitoes using a coaxial-probe technique, realistic FDTD simulations were possible for far field exposure between 2 and 240 GHz. The absorbed RF power *P*_*abs*_ for this insect is lower than for other insects, with a maximum of 5.64 nW for an incident field strength of 1 V/m. Female mosquitoes absorb more power than male mosquitoes, while the body part absorbing most power is the thorax. The distribution of the electric field in and around the mosquito showed a higher field strength in the insect for 120 and 240 GHz than for 6 GHz. The *P*_*abs*_ for EMFs with a frequency of 60 GHz was 16 times larger than for 6 GHz, with the latter frequency the upper limit of current telecommunication networks. For 120 GHz, this increase is even larger compared to 6 GHz, with a factor 21.8. Around this frequency, the maximum in RF EMF absorption was observed for all mosquitoes. In the future, the carrier frequency of telecommunication systems will also be higher than 6 GHz. This will be paired with higher absorption of EMF by yellow fever mosquitoes, which can cause dielectric heating and have an impact on behaviour, development and possibly spread of the insect.

## Supporting information

S1 File STL file of a 3D *A*. *aegypti* male model. A male mosquito mesh 3D model.
(STL)

S2 File STL file of a 3D *A*. *aegypti* female model. A female mosquito mesh 3D model.
(STL)
